# CARE-HOUSE: developing a framework for conceptualizing social implications of digital health technologies in palliative care

**DOI:** 10.1186/s12904-026-02114-z

**Published:** 2026-04-27

**Authors:** Natalie Öhl, Tobias Steigleder, Eva Maria Hille, Matthias Braun, Alina Weisser, Felix Mühlensiepen, Martin Vossiek, Björn Eskofier, Alexander Kölpin, Peter Dabrock, Cynthia C. Forbes, Christoph Ostgathe, Maria Heckel

**Affiliations:** 1 Department of Palliative Medicine, University Hospital Erlangen, Friedrich- Alexander-Universität Erlangen-Nürnberg, Erlangen, Germany; 2 Department of (Social) Ethics, Faculty of Protestant Theology, Rheinische Friedrich- Wilhelms-Universität Bonn, Bonn, Germany; 3 Center for Health Services Research, Faculty for Health Sciences Brandenburg, Brandenburg Medical School Theodor Fontane, Ruedersdorf, Germany; 4 Department of Electrical-Electronic-Communication Engineering, Institute of Microwaves and Photonics, Friedrich-Alexander-Universität Erlangen-Nürnberg, Erlangen, Germany; 5 Chair of Machine Learning and Data Analytics, Friedrich-Alexander-Universität Erlangen-Nürnberg, Erlangen, Germany; 6 Institute for High Frequency Technology, Hamburg University of Technology, Hamburg, Germany; 7 Chair for Systematic Theology II (Ethics), Friedrich-Alexander-Universität Erlangen- Nürnberg, Erlangen, Germany; 8 Wolfson Palliative Care Research Centre, Hull York Medical School, University of Hull, Kingston upon Hull, UK

**Keywords:** Palliative care, Technology, Social implications, Qualitative research, Framework

## Abstract

**Background:**

The use of digital health technologies holds potential benefits for palliative care. Their implementation is fundamentally shifting routines and care practices. These shifts entail social implications that are complex, may emerge gradually and are challenging to identify. However, the social implications of digital health technologies are not conceptualized yet. This study addresses this gap by developing a framework on the social implications of digital technologies in palliative care, where the importance of social inclusion and interpersonal connection is particularly evident.

**Methods:**

We assessed the potential social implications of digital health technologies’ use in palliative care through a sequential research design using qualitative empirical methods of social research. Alongside conducting an iterative narrative literature review, we held multidisciplinary expert consultations using a conceptual mapping method and focus groups involving researchers, clinicians, and a patient and public involvement group, analyzed with qualitative structured content analysis.

**Results:**

Participants as well as the literature review identified key areas to the understanding and analysis of the social implications of digital health technologies in palliative care: principles and objectives of palliative care (patient-centered care, Total care, multiprofessional collaboration, relieving suffering, improving quality of life); the various actors involved in a specific care practice; the different roles they might have; the interactions among different actors; the tasks individual actors might carry out; the processes they are involved in; and the contexts they are embedded in. These factors were summarized and set out in the CARE-HOUSE Framework to conceptualize the social implications of digital health technologies’ use in palliative care and to support researchers in assessing the impacts of these technologies.

**Conclusions:**

The CARE-HOUSE Framework provides guidance for analyzing the social implications of digital health technologies in palliative care. It will be for future studies to evaluate the framework’s adaptability and scalability to diverse technologies and settings.

**Supplementary Information:**

The online version contains supplementary material available at 10.1186/s12904-026-02114-z.

## Background

New digital technologies are gaining increasing prominence in the healthcare context and have potential use within palliative care [1–3]. They include mobile health (mHealth), health information technology, wearable devices, telehealth and telemedicine [4], Artificial Intelligence [5], and the use of sensor-based approaches [6]. Digital health technologies may serve to improve the quality and effectiveness of palliative care services, provide access to care [1, 3], support the coordination and planning of care, assist with decision-making [7], or offer psychosocial support [8].

The implementation of digital health technologies is fundamentally shifting routines and practices in palliative care [9]. This transformation extends far beyond issues relating to the acceptance or adoption of technology; it has the potential to reshape core concepts around the delivery of care, alter established care paradigms, and initiate shifts in deeply-held attitudes about the nature of palliative care itself. These potential social changes are complex and may unleash unforeseen effects which may be delayed in their emergence and difficult to identify [10]. Palliative care represents a unique and complex medical context [11, 12], which entails a strong emphasis on social inclusion and interpersonal interactions. As such, the adoption of digital health technologies in this field may carry distinct social implications that go beyond those generally brought about by processes of incorporating technology into healthcare [13]. Rather, it could reshape the fundamental nature of caregiver-patient relationships, redefine the meaning of “being present” in end-of-life care, and challenge traditional notions of quality in its delivery. Empirical research shows that healthcare professionals perceive a risk that excessive use of digital health technologies might contribute to dehumanization in the practice of palliative care [14]. The inability of digital health technologies to replace the unique human connection that is integral to palliative care is empathized [14]. These concerns reflect deeper questions about how technology might transform not just the practical aspects of delivering care, but also the principles and objectives that are underlying palliative care and are traditionally characteristic of it. Empirical studies have taken place on the ethical, legal, and social implications of digital technologies [10, 15–18]. To our knowledge, however, there is an incomplete understanding of how digital health technologies are changing the design of care, the methods of its delivery, and the attitudes of those delivering and receiving it. There is currently no framework that conceptualizes the social implications of digital health technologies, despite their relevance in palliative care. The project set out in this paper sought to tackle this state of affairs by developing an empirically based framework whose aim is to help investigate social implications of digital health technologies for palliative and end-of-life care.

## Methods

### Design and setting

**Fig. 1:**
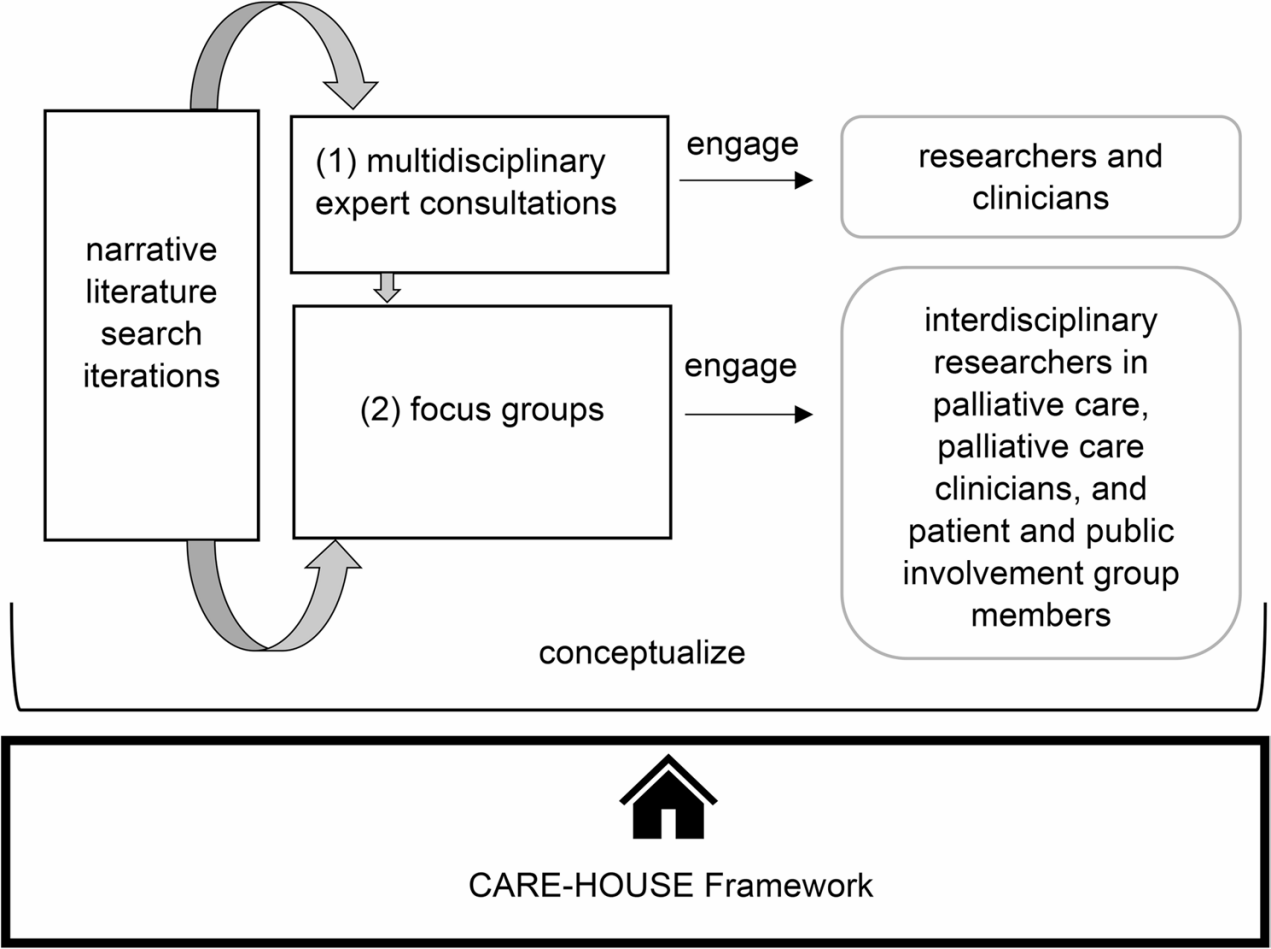
Study design

We developed a sequential qualitative design [19] (9/22 − 7/23) (Fig. 1) relying on a constructivist research approach, with the aim of conceptualizing the social implications of digital health technologies in palliative care. The research proceeded in two stages, (1) multidisciplinary expert consultations and (2) focus groups, which we carried out consecutively and whose findings fed into the development of the CARE-HOUSE Framework. Alongside this empirical research, we carried out an iterative narrative literature review that informed the conceptualization.

We conducted the research in the hospice and palliative care setting within the EmpkinS (German Research Foundation - DFG; CRC 1483) research project carried out at a university hospital in Germany [20]. The study was approved by the local ethics committee, the Ethical Committee of the Faculty of Medicine in Erlangen, on 01.12.2020, 479_20 B.

### Sampling and recruitment

#### Multidisciplinary expert consultations

A sample of ten iterative expert consultations was elected to encompass three to ten participants each. To this end, we identified three relevant stakeholder perspectives (researchers, healthcare professionals, and patient and public involvement group); NO and MH approached potential expert participants personally. We included experts that are eminent scholars from the EmpkinS CRC at Friedrich-Alexander-Universität Erlangen-Nürnberg, Germany and associated institutions that have issued relevant publications in their fields of expertise. The sample included both researchers and clinical professionals from diverse disciplines, including palliative care, theology/ethics, psychology, sociology, medical engineering, and medical informatics. Experts are understood to be experienced and knowledgeable individuals who can identify where digital health technologies could have implications in palliative care. We reconstructed patterns in their descriptions and were able to conceptualize social implications based on these.

#### Focus groups

The same three stakeholder groups were elected for the focus groups in order to obtain critical views from each of these groups throughout the stages of research, development, and implementation of technology and its use in patient care. Participants were recruited by email; we specifically targeted individuals organized in associations in the context of palliative care. Applying the constructivist approach, the group dynamics in the focus groups produced empirically measurable data on collective constructions of meaning, which have consolidated our initial draft.

### Data collection

#### Multidisciplinary expert consultations

Expert consultations were conducted by NO and MH between September 2022 and May 2023 (Table 2). A conceptual map was used to visualize the identified potential parameters for the definition of social implications of digital technologies in palliative care and their interactions with one another as reconstructed by the experts’ statements. The map was expanded with each round of consultation, each of which enabled participants to challenge assumptions and suggest modifications [21].

#### Focus groups

The focus groups took place between May and July 2023 and were conducted by trained facilitators (NO, MH, FM – all researchers in the field of palliative care). They took place either online (F1) or in person at the University Hospital Erlangen (F2, F3); their durations were 75 min (F1, F2) and 45 min (F3) respectively. At the beginning of the focus groups, participants were asked to fill in a short questionnaire (Table 3) to ascertain their gender, age, background/occupation, and technology acceptance. The latter - the attitude to the adoption or use of technology – was assessed using four items from the short scale for “technology commitment” created by Neyer et al. [21, 22]. Each item can be assessed on a 5-point scale ranging from “strongly disagree” to “completely agree”. The sum total of points scored indicates the extent to which the participant has a positive, accepting attitude toward technology (20: highest acceptance).

At the beginning of each focus group, the facilitators raised participants’ awareness of potential social implications of digital health technologies by setting out two examples. Within F1 we used the conceptual map to gain insights on completeness, practical relevance and generalizability of those parameters; F2 and F3 took place in person, which allowed us to use a hospital toy house to provide a sensory visualization of the parameters identified in the expert consultations. A student assistant was present at the focus groups and subsequently transcribed the audio recordings using the transcription guide by Dresing and Pehl [23].

#### Narrative literature review

We conducted a continuous and iterative narrative literature review on topics emerging from expert consultations and focus group discussions to contextualize our empirical findings within existing research discourse and integrate documented observations from the literature into our conceptual framework.

A narrative approach was chosen to integrate heterogeneous empirical and conceptual literature relevant to palliative care and the social dimensions of digital health technologies. Two researchers (NO, MH) developed and continuously refined the search strategy, including search terms and databases, guided by insights from the consultations and discussions. The search was conducted by a student assistant between September 2022 and November 2023 across the following databases: PubMed, MEDLINE, Scopus, Google Scholar, Livivo, Wiso-net, and Springer.

Search terms were used in combination with “palliative care” and/or “patient” and/or “population” and included: quality of life; wellbeing; symptom control; symptom burden; suffering; needs; skills; values; vulnerability; biography; experiences; autonomy; pain; suffering; expectations; wishes; individuality; diversity; privacy; emotions; spirituality; personality; resilience; acceptance; participation; cognitive impairment; patient will; culture; burden; professionals; physicians; doctors; bereaved; interdisciplinarity; human-interaction; human-technology interaction; humanity; humanness; emotional care; human interaction; interpersonal care; communication; conversation; relationships; human relations; information; (shared) decision-making; therapy; treatment; self-conception; self-imaging; roles; hierarchies. All search terms were applied in both German and English.

### Data analysis

#### Multidisciplinary expert consultations

We used conceptual mapping with an adapted form of focus group illustration maps [24] to visualize the data collected within the multidisciplinary expert consultations on a Miro Board [25]. Within each consultation, NO and MH refined the conceptual map using inductive categorization. The elements were organized into thematic clusters, with additional categories, subcategories, examples, and variations incorporated. Relationships between the elements were also graphically depicted to enhance understanding.

#### Focus groups

The focus groups were analyzed using the method of qualitative structured content analysis [26] and MAXQDA software [27]. First, two coders (NO and a student assistant) carried out deductive coding based on the parameters from the conceptual map, before the coding scheme was adapted through inductive coding and the addition of subcodes and sub-subcodes (Table 1). To attain as high a level of intersubjectivity as possible and to verify the categories, the coding was tested and discussed with NO and MH in two feedback loops. The adaptation of the coding scheme was an iterative process which involved going through the material multiple times (Table 1). Intersubjective differences in interpretation were minimized by agreeing on clear definitions of codes (coding guidelines).

**Table 1:** Coding scheme for the focus groups (* = inductive codes added after deductive coding)

codes (deductive)	subcodes (deductive/inductive*)	sub-subcodes (inductive*)
principles and objectives of palliative care	patient-centered care	
	total care	
	multiprofessional team	
	quality of life	
	human connection*	
actors and characteristics	patients	life circumstances*
		skills and knowledge*
		burdens and challenges*
		needs and preferences*
	informal caregivers	life circumstances*
		skills and knowledge*
		burdens and challenges*
		needs and preferences*
	healthcare professionals	life circumstances*
		skills and knowledge*
		burdens and challenges*
		needs and preferences*
	technology	purpose*
		functions*
		quality*
		use*
		design*
		values*
delivery of care	interactions	interactants*
		purpose*
		modalities*
	roles	self-conception*
		perception of others*
		determinants*
	tasks	actors tasked*
		details of task*
		extent of resources required*
	processes*	points of intersection*
		procedures*
		outcomes*
context	societal	demographic factors
		economic factors
		political and policy factors
		law
		culture*
		ethics*
	organizational	infrastructure
		technical facilities
		staff structures
		management
		funding
	professional	standards
		guidelines
		quality criteria
		science
	structural*	location-related factors*
		healthcare-systems and services*
		networks*
		services available*

#### Narrative literature review

We included empirical and theoretical publications addressing social, ethical, emotional, or experiential aspects of palliative care and also their relation to digital health technologies. Studies focusing exclusively on clinical outcomes were excluded. Titles and abstracts were screened for relevance and the identified literature was thematically categorized, tabulated, and reviewed by both researchers (MH, NO). Key insights were discussed and integrated into the evolving conceptual framework. A total of 60 publications were included in the final synthesis, which followed a thematic approach to identify recurring concepts and relationships. The synthesized findings of the narrative literature review formed the theoretical foundation for conceptualizing the social impliciations.

Following a sequential research approach, we synthesized the insights emerged from the multidisciplinary expert consultations, the focus groups and the narrative literature research to conceptualize social implications of digital health technologies in palliative care. This process culminated in the CARE-HOUSE Framework.

## Results

### Sample characteristics

#### Multidisciplinary expert consultations – participants characteristics

We conducted ten multidisciplinary expert consultations including experts with a broad range of expertise (Table 2).

**Table 2:** Participants in and dates of multidisciplinary expert consultations

Date	participants´ background
September 19, 2022	palliative care professionals researchers in the disciplines of medical engineering, psychology, sociology
September 20, 2022	researchers in the disciplines of psychology and medical information technology
September 26, 2022	researchers in the disciplines of theology/ethics
November 10, 2022	members of a patient and public involvement group
February 15, 2023	researchers in the disciplines of medicine, psychology, engineering
March 1, 2023	researchers in the disciplines of theology/ethics
March 3, 2023	researchers in the disciplines of palliative care, health services research, medicine
April 13, 2023	palliative care professionals
May 11, 2023	researchers in the disciplines of theology/ethics
May 15, 2023	researchers in the disciplines of health services research in palliative care

#### Focus groups: participants’ characteristics

We conducted three focus groups involving a total of 18 participants. The group of researchers and professionals in palliative care (*n* = 7) primarily consisted of individuals aged 31 to 40, with a slight majority being female. Informal caregivers and interested citizens (*n* = 5) were predominantly over 60 years old and mostly female. Clinical professionals (*n* = 6) were aged 41 to 60, with an equal gender distribution. The mean technology acceptance across all three groups was similar (Table 3).

**Table 3:** Characteristics of focus group participants

Group	researchers and professionals in palliative care (F1)	informal caregivers of palliative care patients and interested citizens (F2)	clinical professionals in palliative care (F3)
Participants	*n* = 7	*n* = 5	*n* = 6
Age	< 20 years 0	< 20 years 0	< 20 years 0
	20–30 years 1	20–30 years 1	20–30 years 0
	31–40 years 4	31–40 years 0	31–40 years 0
	41–50 years 2	41–50 years 0	41–50 years 2
	51–60 years 0	51–60 years 0	51–60 years 4
	> 60 years 0	> 60 years 4	> 60 years 0
Gender	male 2	male 1	male 3
	female 5	female 4	female 3
	others 0	others 0	others 0
Background/occupation	researcher 2	engineering 2	medicine 3
	medicine 2	psychology 1	administration 2
	psychology 1	education 1	physiotherapy 1
	administration 1	administration 1	
	no information 1		
Technology acceptance	range: 9–18	range: 12–15	range: 10–19
	mean: 13.6	mean: 13.8	mean: 13.5

### Conceptualizing social implications of digital health technologies in palliative care

Participants of the expert consultations and focus groups and the findings of the narrative literature review identified the following key factors as relevant to the understanding and analysis of the social implications of digital health technologies in palliative care: principles and objectives of palliative care; the various actors involved in a specific care practice; the different roles they might have; the interactions among different actors; the tasks individual actors might carry out; the processes they are involved in; and the contexts they are embedded in. The use of digital health technologies might influence each of these dimensions of palliative care practice, which together informed the development of the CARE-HOUSE Framework.

### The CARE-HOUSE Framework

**Fig. 2:**
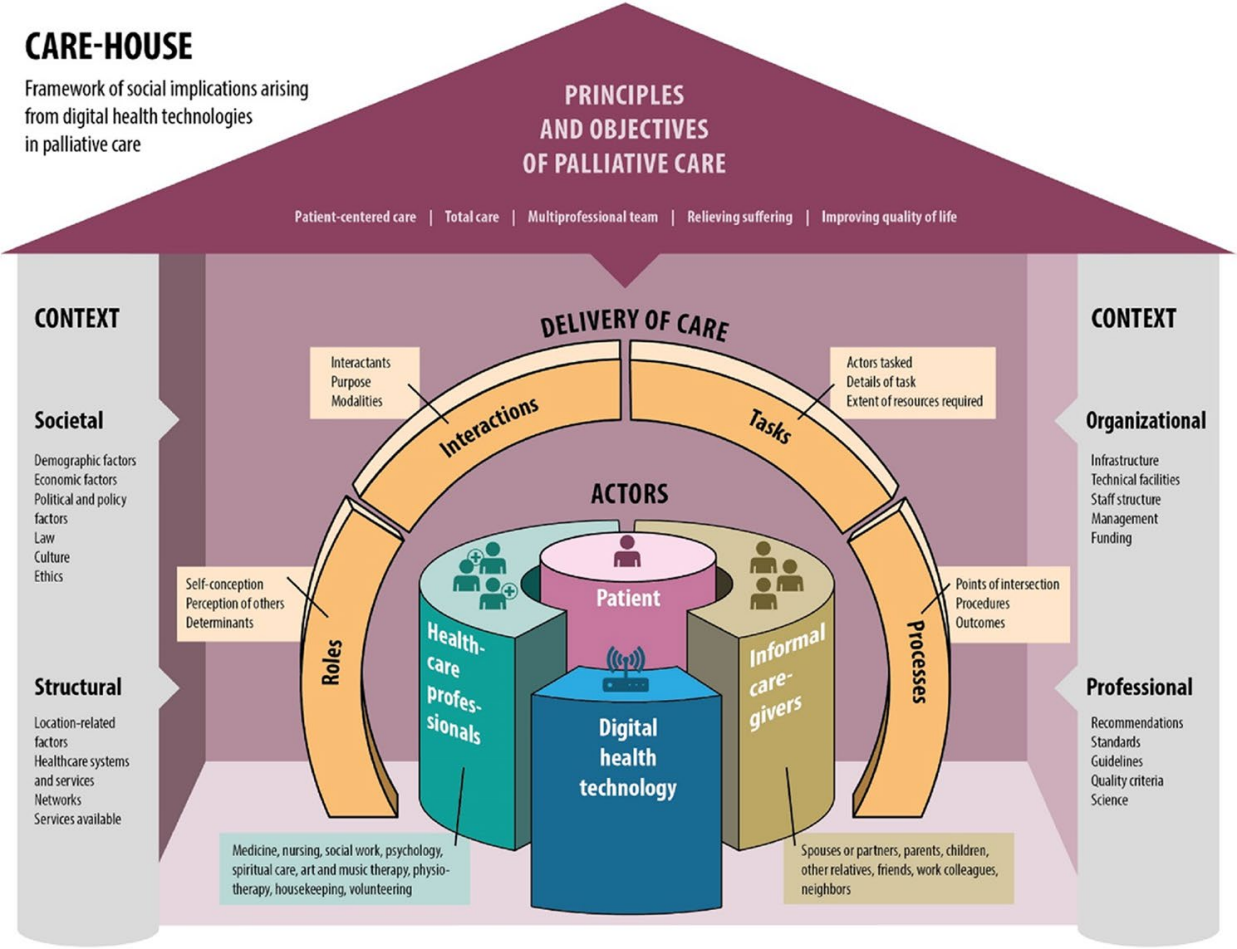
CARE-HOUSE Framework, conceptualizing social implications of digital health technologies in palliative care

In creating the CARE-HOUSE Framework, we conceptualized the social implications of technology use in palliative care as the overall picture of the impact that all actors attribute to the effects of a particular digital health technology in question on the practice of palliative care (Fig. 2). Depicted as a metaphorical house, CARE-HOUSE contains various components and factors that interact dynamically, influencing one another reciprocally [11]. The framework’s purpose is to help its users identify areas and issues for consideration. Guided by CARE-HOUSE, researchers can assess and generate hypotheses about the impacts of these technologies and use them to inform processes of data collection and analysis. A further purpose of the framework is for supporting the consideration of potential benefits, risks, and uncertainties when evaluating the implications of technology use in the palliative care setting, drawing throughout on the principles and objectives underlying palliative care. Its design enables its adaptation over time, reflecting changes in technology use and care practices [28]. Its component categories were derived from the focus group coding scheme (Table 1) and the multidisciplinary expert consultations we conducted.

### Principles and objectives of palliative care

The principles and objectives of palliative care serve as a benchmark for assessing the impact of digital health technologies in this area; the task here is to determine whether these technologies support or detract from the core principles of palliative care as defined by the WHO in 2002 [29, 30], which include patient-centered and total care, multiprofessional collaboration, a focus on quality of life, and the relief of suffering. When introducing digital health technologies in specific palliative care settings and situations, the application of these principles requires awareness, and therefore conceptualization, of the social implications attached to these technologies, with the aim of ensuring that technological interventions are in line with the essential objectives of palliative care.

#### Patient-centered care

Palliative care follows a patient-centered approach, aligning treatment and care planning with the individual wishes, goals, aims, preferences, backgrounds, values, and needs of patients and their informal caregivers [31]. It respects the uniqueness of each patient and their family and is accessible to all patient populations, including all ages, diagnostic categories, and settings [32].

#### Total care

Palliative care follows the holistic total care approach [33], taking into account not only the patient’s physical condition, but the whole person, including his or her experience of the illness [34]. Building on Cicely Saunders´ total pain concept [35, 36], the idea of total suffering, to which total care is a response, is understandable as a complex phenomenon, incorporating mental, social, and spiritual alongside physical factors.

#### Multiprofessional collaboration

Addressing the varied and complex issues that emerge in the process of palliative care requires a range of competencies and skills. For this reason, palliative care is provided by a multiprofessional team comprising representatives of various different professions or disciplines [37].

#### Relieving suffering

Participants pointed out that patients with life-threatening diseases may suffer from severe symptoms. Palliative care aims to prevent and relieve burdens imposed by the illness and its treatment [32].

#### Improving quality of life

The core aim of palliative care is to improve, maintain or enhance the best possible quality of life for patients and their informal caregivers [31, 32]. Palliative care therefore expands traditional models of disease and focuses on quality of life, regardless of the stage of the patient’s disease [32].

### Actors

All people that are affected in any way by technology are considered as actors; participants of the focus groups stated that this includes people that do not interact directly with technology, or may not even be aware of its use. There are typical groups of actors with salient characteristics (see Table 4); however backgrounds and attributes might distinct subgroups [9].

**Table 4:** Actor characteristics with relevance to the evaluation of social implications associated with technology use in palliative care

	patients and informal caregivers
Life circumstances	life course-related factors
	cultural influences
	individual factors (personality, beliefs, interests, views) [38]
	personal resources (resilience, confidence)
	social connectedness (participation in the life of society, relationships) [39]
	caregiver situation (setting [40], geographical proximity, other caring responsibilities, duration of care, relationship to the patient [41])
	occupation
Skills and knowledge	educational background
	cognitive abilities (cognitive impairment, ability to understand medical information) [42]
	e-health literacy (ability to process information, ability to use digital services, ability to think critically and logically, computer literacy) [14, 43]
	state of health (comorbidities) [28, 40]
	physical abilities [28]
	body perception (integrity of the body) [44]
	knowledge base
Burdens and challenges	emotional and mental stress/distress (grief, helplessness, fatigue and overload, worries and uncertainty) [45]
	patient’s physical symptoms (pain, delirium, lack of energy, fatigue, dyspnea, constipation, nausea, vomiting) [31, 46] informal caregiver’s physical symptoms (distress) [41]
	social challenges (loneliness, not feeling understood, sense of loss of control over their life) [45]
	financial challenges [41]
Needs and preferences	desires, expectations, visions and aims in terms of treatment, care and beyond (privacy, autonomy, dignity, religion and spirituality) [39, 47]
	values
	information needs (openness, transparency and honesty, understandable and comprehensive information) [38]

	healthcare professionals
Skills and knowledge	educational background (qualifications)
Burdens and challenges	emotional and mental stress (grief of patients and informal caregivers, frustration and helplessness) [42, 48, 49]
	workload (unpredictable work, time pressure, time constraints) [42, 48]
	wish to provide good patient care (taking time, meeting holistic needs) [42]
Needs and preferences	preferences regarding technology [14], care and beyond

#### Patients

Palliative care patients typically are incurably ill and have symptoms caused by their condition [30]. They are having specific characteristics, features and attributes within the domains of the total pain concept [32, 35, 36].

#### Informal caregivers

Palliative care includes informal caregivers, such as patients´ spouses, parents, children, siblings, other relatives, friends, work colleagues, or neighbors [41], as recipients of care. They are described as “hidden patients” and at the same time are regarded as being part of the palliative care team, carrying a dual role [41, 50].

#### Healthcare professionals

One defining characteristic of palliative care is its multiprofessional team, which - depending on the setting – may include physicians, nurses, psychologists, social workers, physiotherapists, professionals providing spiritual or pastoral care, and volunteers [33, 37].

#### Characteristics

Understanding the social implications of technology use in palliative care requires consideration of the life circumstances, skills and knowledge, burdens and challenges, and needs and preferences of a group of actors (Table 4).

#### Digital health technologies

Digital health technologies are potential additional actors, but may also serve as tools [51]. Participants of the focus groups described digital health technologies´ purposes, functions, quality, use, design, and values, considering these to be characteristics of digital health technologies with relevance to the analysis of their social implications.

##### Purpose

Digital health technologies are developed and implemented for a specific reason and with a particular aim and intended purpose. They typically have a value proposition relating to specific use cases and healthcare scenarios [28]. On the user side, being aware of the value of a technology and feeling it is beneficial can lead to greater adoption [43]. The intentions and motivations of actors [52, 53], including the technology’s perceived usefulness [54], are known to be relevant to actors’ engagement with and acceptance of a technology.

##### Functions

Digital health technologies generate a certain kind of knowledge [28]. They offer technical functions, which can be adaptable to individual needs, and include for example access to support [43].

##### Quality

The system, information and service quality [52, 53, 55] of digital health technologies and the stage of their development influence the quality and safety of their use.

##### Use

Human actors use digital health technologies and therefore need to handle and interact with them. The complexity and usability of technologies are of relevance in this regard, as is users’ possession or otherwise of the extent of digital literacy necessary for the use of a digital health technology [28]. A technology’s perceived ease of use can similarly affect the extent of its acceptance [54]. However, it also has to be acknowledged that not all actors will want to use technology [43].

##### Design

Relevant aspects of a technology’s design include “feel”, visual appearance, the user interface, and graphic design. Visual appearance may be of less significance where users do not necessarily catch sight of technology on a regular basis; one example here might be radar-based sensors placed under a user’s mattress [56].

##### Values

Recent years have seen intense debate around whether it is possible to describe technology as value-neutral. The overall consensus, notwithstanding variations in detail, is that technology is strongly connected to moral values. Digital health technologies accordingly carry values, which require consideration when evaluating their social implications. Focus group participants particularly highlighted control over technology and the importance of human connection, which latter they regarded as a core principle of palliative care. Values participants cited as relevant included security, in terms, for example, of data security or data ownership, and the trustability of a technology [43].

### Delivery of care 

Part of the analysis of technology’s social implications entails assessing the influence exerted by the use of digital health technologies on the delivery of care, in its various aspects, and examining how the principles and objectives of palliative care are upheld. The delivery of care comprises interactions, roles, tasks, and processes.

#### Interactions

When examining social implications of digital health technologies, participants in the expert consultations and focus groups stated that it is crucial to identify the relevant interactions – that is, behaviors and actions – that take place among actors in a specific care scenario, and to understand how those interactions are characterized. Digital health technologies have the potential to create new types of interactions and affect existing ones. The description of interactions can use categories as follows:

##### Interactants

Interactions can take place between two or more human beings from the same group of actors or from different ones (human-human interaction). Where digital health technology is regarded as an actor, interactions can also take place between humans and technology (human-technology interaction).

##### Purpose

The purpose of interactions might be the sharing of information, the satisfying of needs for information, decision-making on issues such as advance care planning, or the discussion of medical options and the aims of care [57]. Care activities may also be the purpose of an interaction, and can encompass diagnosis, therapy, nursing, monitoring, and emotional and psychosocial care according to the consulted experts. In the delivery of palliative care, developing a personal relationship with patients and informal caregivers is essential, and this may also be an interaction’s purpose. The purpose of an interaction is often related to its context and to the roles or tasks an actor has. Interactions with digital health technologies might serve additional purposes, such as documentation, monitoring, or the conduction of diagnostic processes.

##### Modalities

Interactions between actors can be verbal or non-verbal. Important characteristics of verbal interactions include the language and speech patterns used, which may, for example, seek to adapt to the patient’s own language and their level of understanding [58, 59]. An interactant’s share of the total speech in the interaction and their other speech behaviors, such as listening, may also be of relevance [58, 59]. Further aspects of interaction that merit analysis are the communication channels used and any barriers to communication; some patients, for example, are unable to communicate verbally. Finally, it may be important to take note of attitudes within the conversation, which may manifest as honesty, empathy, attentiveness, respect and taking the patient seriously [45, 58, 59]. Aspects of nonverbal interaction include body language, eye contact, position in the room, and physical posture [58]. They also include physical touch, such as massages, stroking or holding, which can express empathy and support, particularly if verbal communication is limited or no longer possible [60]. Interactions are influenced by various factors, such as frequency. Palliative patients and their informal caregivers place particular importance on their treating healthcare professionals being present with them and having time for them [42, 45]. Familiarity may be a further significant factor here; participants in one focus group (F3) stated that trust and a sense of security may emerge from a professional becoming a familiar face or voice to a patient. The modalities of an interaction are often influenced by an actor’s role.

#### Roles

Participants of the focus groups considered roles to denote the positioning of actors in the social context surrounding the practical delivery of care, including professional and personal roles of relevance to care. One actor may hold several different roles simultaneously. Clarity on professional roles is an important factor in the provision of care [57]. The use of digital health technologies may affect the roles of actors; this is one of the social implications of digital health technologies’ use in palliative care, as is the technologies’ potential influence on how actors see themselves and other actors.

##### Self-conception

The consulted experts stated that human actors have a self-conception, that is, an idea of themselves and an understanding of their role. Healthcare professionals have a professional self-conception; for example, palliative care nurses will largely see themselves as advocates for patients within the interdisciplinary team, because of their comprehensive view and holistic knowledge of patients [42, 57]. Healthcare professionals in general consider themselves as partnering with patients and families in palliative care [39]. Palliative care physicians may see themselves as companions of the patient [61]. Patients likewise have self-conceptions which represent their understanding of their own role; patients receiving palliative care see themselves as experts on their own condition and as autonomous individuals. Participants of one focus group (F2) also stated patients may have difficult self-conceptions, such as viewing themselves as victims of their disease. Digital health technologies could challenge patients’ self-concepts and their perceptions of their bodies [44].

##### Perceptions of others

The consulted experts noted that, alongside their self-conception, human actors have a perception of other actors and their roles, which does not always align with the other actor’s self-conception, potentially causing conflict to arise. Actors have specific expectations toward other actors on the basis of their perceptions of the other’s role. In palliative care contexts, informal caregivers may perceive patients as being in need of assistance. If this perception does not align with the patient’s own self-conception it may lead to patients feeling disrespected [38]. Where digital health technology is considered as an actor, it also fills a role, and other actors will have a certain perception of its role. Human actors may, for instance, consider digital health technologies to be in the role of beneficial “enablers” [62] or enhancements to care [14]. Technology can fulfill uncomplicated tasks [63]; it can either be a supplement to or a substitute for human care. Actors may state what roles they do not want technology to fulfil, such as replacing the human delivery of care [14].

##### Determinants

The role of an actor is highly determined by the societal and professional conditions in which they are operating according to the consulted experts. The general reputation of physicians within society and healthcare, for example, can influence the way physicians see themselves and their own role, but also how other actors see their role. A society’s image of illness and frailty can act as a determinant to the role of patients. Besides, actors’ positions in social networks of relationships can determine their roles; an informal caregiver may, for instance, be in the role of a daughter who feels responsible for her parents and does not want them to decline. Family members of palliative care patients can act as informal caregivers [42], but at the same time they are themselves in need of care and receive care from professional caregivers [13]. An actor’s socialization, in terms of their life story, education or demographic characteristics, has a powerful influence on their role, as does any authority they may hold in a specific situation; family members can have legal authority or power of attorney and therefore act as proxy decision-makers for patients [42]. Authority also comes in the form of knowledge and expertise, as held by healthcare professionals, especially physicians, who can decide on medical treatment options. As patients and informal caregivers are often medical laypeople, there is an asymmetrical power relationship and hierarchy, and physicians may act as gatekeepers [64] to medical services. The competences are often linked to the tasks an actor has, and therefore also the tasks can determine a role an actor is having.

#### Tasks

Tasks within the practical delivery of care encompass activities conducted by various actors and include tasks that healthcare professionals carry out and responsibilities supporting the care process and pertaining to informal caregivers and patients. These tasks are usually part of larger processes within the practice of care. Focus group participants noted that digital health technologies may affect the conduction of tasks, including who does what and to what extent. An effective evaluation of the social implications surrounding digital health technologies should involve consideration of whether their use affects the following factors:

##### Actors tasked

An actor completing a task may be human or a technology. According to the consulted experts, tasks are often related to the role of an actor, as the role comes with particular responsibilities, authority, and expectations. Some tasks are shared by multiple actors.

##### Details of task

This domain includes which tasks are carried out by specific actors and what each step or stage of the task looks like; it is closely linked to processes, as individual tasks are usually part of larger procedures. The palliative care process, from healthcare professionals’ point of view, entails treatment planning, assessing and monitoring the patient’s state of health [65], patient education [66], coordinating care, and providing access to religious ritual where desired [67]. Informal caregivers, by contrast, may be responsible for coordinating care or assisting with physical care tasks [41]. Each of these tasks consists of various stages of work and individual activities.

##### Extent of resources required

Tasks vary in their extent and their completion requires particular resources. Resource intensity is related to the number and complexity of stages involved in the task, the time required, and the physical and emotional effort that goes into the task. Participants of the focus groups stated that the use of digital health technologies can affect the extent of resources required for a task within the delivery of care.

#### Processes

Processes are definable as connected and consecutive tasks with a specific overarching aim. Processes may take place on different levels, such as within a single ward, within an organization, or within the healthcare system in general. In terms of patient-centered healthcare, processes are often aligned to the patient and their individual or disease-related care pathway [68]. Processes are closely connected to and dependent on the conditions prevailing within a specific profession or organization. Digital health technologies can affect the interfaces and procedures involved in processes and their outcomes according to focus group participants. The introduction of digital health technologies to healthcare settings necessitates consideration of the extent of change to organizational routines needed, and of the planning, implementation, and monitoring of change [28].

##### Intersections

Processes involve the collaboration of actors and the combination of the tasks carried out by each of them. They will therefore include transitions among actors and the functional, organizational and personal interfaces between them [69]. Processes may also entail interaction with external actors, such as health insurers. Social implications may also affect some actors who are not directly involved in a process, but still affected by it. Interactions and intersections may create issues such as information loss or uncertainties around responsibilities [69], which are related to the roles of actors.

##### Procedures

Procedures, as parts of processes, are structured combinations of individual tasks. Procedures can be standardized and may involve certain organizational requirements. Input variables to procedures, include resources, such as staff and materials, which a process transforms into output variables [68, 69].

##### Outcomes

Processes have a defined goal, their outcome, whose attainment is available to evaluation on the basis of defined measurement variables and target variables [69]. The outcome of a process within palliative care is ideally evaluated in terms of the principles and objectives governing palliative care in general.

### Context

The context around the practical delivery of care frames it and influences what happens within it; it depends on the specific palliative care setting involved, on whether, for example, it is an in- or outpatient setting. Contexts can change over the course of time, for example due to changes in legislation or the introduction of new care standards, and there can also be reciprocal influences of the practice of care on the context.

#### Societal

The societal context refers to the circumstances, processes and developments within a society that influence healthcare and therefore palliative care. Society is made up of people, each carrying a set of influences including demographics and cultural settings; political and policy factors are likewise material within a society, as are laws and legislative and economic factors [28, 55, 70]. Similarly, moral values are significant in a society, which will seek consensus on which moral values it recognizes; some with particular relevance to palliative care are autonomy, beneficence, not causing harm, and justice [71–73]. Consideration of a societal context can help researchers to analyze and understand both the implicit, taken-for-granted moral values and the institutionally accepted norms that are at work in a particular situation. This may be of heightened importance in the context of palliative care, due, for example, to potential marked differences in how people, and therefore societies, conceive of health and illness, a “good life,” or a “good way to die”.

#### Structural

The concept of structural context denotes the local and spatial environment of a specific practice of care and the system into which it is incorporated and in which it takes place. The structural context encompasses location-related factors, such as whether the care is delivered in an urban or a rural area, alongside other relevant healthcare systems and the scope of services available, such as general practitioners, specialist physicians, outpatient services, hospices, and volunteers, and the networks that exist among various services and organizations [28, 55].

#### Organizational

The organizational context references existing circumstances and conditions within a healthcare organization. Alongside its infrastructure, such as access to equipment and information [43], and technological facilities, such as software and the interoperability of systems [43], an organizational context comprises staffing, staff structures, healthcare professionals´ qualifications, and management practices such as strategy, leadership, and communication [55]. Overarching organizational cultures, particularly the organization’s willingness and openness to innovations, are an additional factor of relevance [28, 55, 70], as is funding, in terms of the financial resources available and, for example, ownership of intellectual property in technologies [28].

#### Professional

The professional context engages existing recommendations, standards, guidelines, quality criteria prescribed by official bodies, evidence derived from research, and knowledge, each of which seek to ensure that care takes place to a high standard of quality.

## Discussion

We provide the CARE-HOUSE Framework that conceptualizes the dimensions of social implications of digital health technologies in palliative care. The CARE-HOUSE Framework provides a holistic model for considering the impacts of digital health technologies on social aspects of palliative care and enabling a response to them, helping researchers to plan, conduct and interpret their work.

In the process of developing the framework, we made use of group discussions with researchers and clinical professionals from the fields and disciplines of palliative care, theology/ethics, psychology, sociology, medical engineering, and medical information technology, alongside a thorough literature review, to enhance its comprehensiveness and robustness. The theories, models and frameworks they referenced [28, 43, 51–55, 70, 74–76] supply solid theoretical foundations for key factors of CARE-HOUSE. The study’s inclusion of members of a patient and public involvement group was key to making sure we took a needs-based approach [77]; their insights and lived experiences played a vital role in informing the framework to make it effective in addressing the specific concerns and priorities of stakeholders in the delivery of care. Expert consultations and focus group discussions were conducted in sequence, complemented by a simultaneous narrative literature review. The CARE-HOUSE Framework emerged iteratively throughout this process, with insights from each step contributing to its development. However, this integrated approach may limit the traceability and transparency of the results for each individual component.

While the framework originally came into being within a specific research project [20] and focused on a single research center, we believe it holds potential for broader application across various types of technology and palliative care settings. We will conduct future case studies [78] to further evaluate the framework’s adaptability and scalability to diverse environments, and may highlight additional limitations of the framework or give rise to proposals for modifications that appear necessary to its wider implementation.

Various methods and tools can support the analysis of social implications attached to the use of technologies. They include the Partnership Canvas [79], the Ethics Canvas [80], the MEESTAR Workshop [81], Acceptance-Risk Workshops [82], and the Q-Sort Technique and Q Methodology [83]. Further research could usefully link these methods to the CARE-HOUSE Framework and investigate their suitability for analyzing social implications of digital health technologies for various different target groups and purposes.

It may prove helpful going forward to create a tool to support the application of the framework and describe ways of empirically assessing and analyzing the various factors that comprise the CARE-HOUSE.

## Conclusion

The CARE-HOUSE Framework provides guidance with the analysis of potential social implications associated with digital health technologies in palliative care. It enables users to recognize and describe changes in conceptions of palliative care and methods of its delivery, as well as taking account of shifts in attitudes among patients, informal caregivers and healthcare professionals. We hope the framework will support the sensitive management of these social implications within technology development and promote the socially conscious implementation of digital technologies in palliative care, consistently centering the core principles of palliative care.

### Implications for research

Future research should focus on validating the CARE-HOUSE Framework´s applicability, usability and generalizability across different types of digital health technologies and care settings. Empirical studies are needed to refine and substantiate the relationships between the framework´s elements, and to identify which aspects are most influential in shaping social experiences in palliative care. Special attention should be given to interactions, as they are central to understanding social implications.

### Implications for practice

The CARE-HOUSE Framework can support practitioners and technology developers in anticipating and addressing potential social impacts of digital health technologies. By examining how technologies affect the framework’s key elements, stakeholders can help ensure that technology integration remains consistent with the core principles of palliative care.

## Supplementary Information

Supplementary Material 1.

## Data Availability

The datasets used and/or analyzed during the current study are available from the corresponding author on reasonable request.

## Abbreviation

ELSI: ethical, legal, and social implications
