# Supplementary material for: CARE-HOUSE: developing a framework for conceptualizing social implications of digital health technologies in palliative care
Source: BMC Palliat Care. 2026 Apr 27;25:116. doi: 10.1186/s12904-026-02114-z (PMC13112699; doi:10.1186/s12904-026-02114-z)
Supplement: Supplementary file 1 — Supplementary Material 1. [file 12904_2026_2114_MOESM1_ESM.docx]

**Supplemental material**

In the narrative literature review the following search terms were used in combination with “palliative care” and/or “patient” and/or “population”: quality of life; wellbeing; symptom control; symptom burden; suffering; needs; skills; values; vulnerability; biography; experiences; autonomy; pain; suffering; expectations; wishes; individuality; diversity; privacy; emotions; spirituality; personality; resilience; acceptance; participation; cognitive impairment; patient will; culture; burden; professionals; physicians; doctors; bereaved; interdisciplinarity; human-interaction; human-technology interaction; humanity; humanness; emotional care; human interaction; interpersonal care; communication; conversation; relationships; human relations; information; (shared) decision-making; therapy; treatment; self-conception; self-imaging; roles; hierarchies. The search terms were used in both German and English.
